# Expression of yeast lipid phosphatase Sac1p is regulated by phosphatidylinositol-4-phosphate

**DOI:** 10.1186/1471-2199-9-16

**Published:** 2008-01-28

**Authors:** Andreas Knödler, Gerlinde Konrad, Peter Mayinger

**Affiliations:** 1 Division of Nephrology and Hypertension, Oregon Health & Science University, Portland OR 97239, USA; 2 Zentrum für Molekulare Biologie der Universität Heidelberg (ZMBH), 69120 Heidelberg, Germany

## Abstract

**Background:**

Phosphoinositides play a central role in regulating processes at intracellular membranes. In yeast, a large number of phospholipid biosynthetic enzymes use a common mechanism for transcriptional regulation. Yet, how the expression of genes encoding lipid kinases and phosphatases is regulated remains unknown.

**Results:**

Here we show that the expression of lipid phosphatase Sac1p in the yeast *Saccharomyces cerevisiae *is regulated in response to changes in phosphatidylinositol-4-phosphate (PI(4)P) concentrations. Unlike genes encoding enzymes involved in phospholipid biosynthesis, expression of the *SAC1 *gene is independent of inositol levels. We identified a novel 9-bp motif within the 5' untranslated region (5'-UTR) of *SAC1 *that is responsible for PI(4)P-mediated regulation. Upregulation of *SAC1 *promoter activity correlates with elevated levels of Sac1 protein levels.

**Conclusion:**

Regulation of Sac1p expression via the concentration of its major substrate PI(4)P ensures proper maintenance of compartment-specific pools of PI(4)P.

## Background

Phosphorylated derivatives of phosphatidylinositol, collectively called phosphoinositides, play essential roles in a wide range of cellular processes situated at intracellular membranes [1]. Recent evidence indicates that phosphoinositides are not only short-lived signals that activate downstream regulatory networks, but also play constitutive roles in organelle identity and membrane dynamics [2]. A key property of individual phosphoinositides is their precisely regulated compartment-specific localization [2,3]. The control and maintenance of diverse intracellular phosphoinositide pools is achieved through the functional interplay of specific sets of lipid kinases and phosphatases. Although it has been established that deficiencies in certain lipid phosphatases can lead to severe human disease [4], it is unknown as to how the expression of these enzymes is regulated. In contrast, the transcriptional regulation of enzymes involved in the biosynthesis of major membrane phospholipids is well characterized [5]. The cellular concentrations of metabolic intermediates required for phospholipid biosynthesis, such as inositol, choline and phosphatidic acid, determine the levels of expression of their respective biosynthetic enzymes [6,7]. However, whether the expression of lipid phosphatases and kinases is controlled by similar mechanisms remains unclear.

The polyphosphoinositide phosphatase Sac1p is a major regulator of PI(4)P levels at the endoplasmic reticulum (ER) and Golgi [8-10]. The precise distribution of PI(4)P between these two organelles is critical for coordinating cell growth with the secretory pathway [11]. Here we show that the cellular levels of yeast Sac1p are regulated at the transcriptional level. We have identified a novel 9-bp element within the *SAC1 *promoter region that is necessary for the regulation of promoter activity. Furthermore, we demonstrate that intracellular levels of PI(4)P correlate with Sac1p protein levels.

## Results

### Identification of promoter elements for regulation of *SAC1 *expression

**Figure 1:**
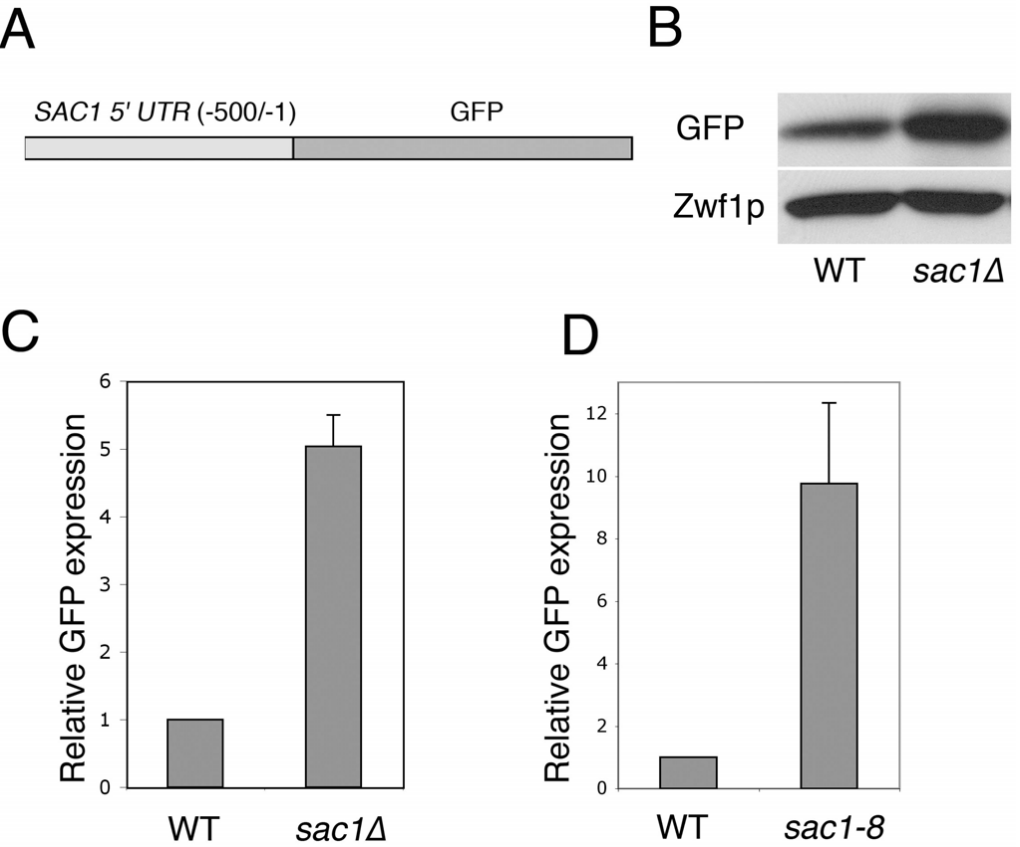
**Elevated activity of the *SAC1 *promoter in a *sac1 *mutant background**. **(A) **Diagram depicting a reporter construct used to examine expression activity in the yeast *Saccharomyces cerevisiae*. The 5'-UTR of *SAC1 *ranging from bp -500 to -1 was fused to the open reading frame of GFP. **(B) **Expression from the GFP reporter constructs. Wild-type and *sac1*Δ yeast cells transformed with a *CEN*-based plasmid containing the *SAC1*(-500/-1)-GFP fusion construct were grown to early log phase at 30°C. Cell extracts were analyzed by SDS-PAGE and immunoblotting using anti-GFP and anti-glucose-6-phosphate dehydrogenase (Zwf1p) antibodies. **(C, D) **Quantitation of relative GFP expression levels in *wild-type, sac1*Δ **(C) **and *sac1-8 ***(D) **strain backgrounds. Data are from at least three independent experiments (+/-SE).

**Figure 2:**
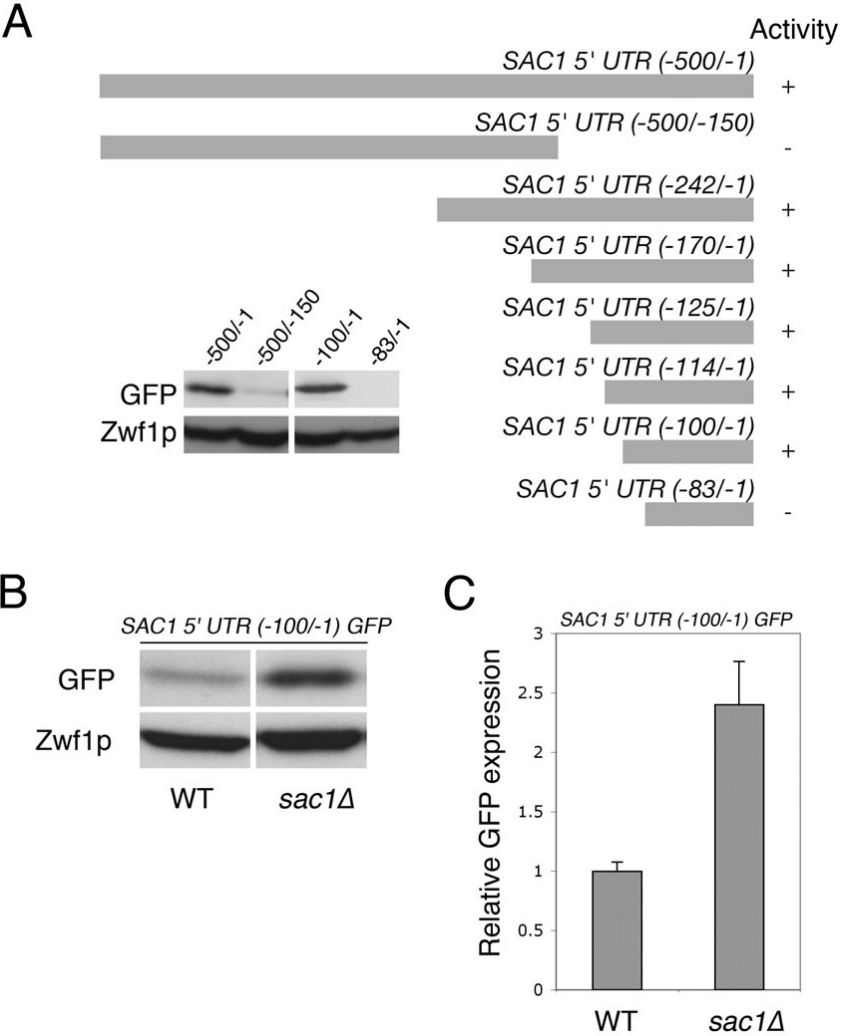
**Characterization of a minimal *SAC1 *promoter region sufficient for regulated expression**. **(A) **Diagram depicting deletion constructs. The constructs were fused to the open reading frame of GFP in a *CEN*-based vector. The plasmids were introduced into a wild-type strain background and promoter activity determined by measurement of relative GFP expression levels in cell extracts. **(B) **Expression of the GFP reporter. Wild-type and *sac1*Δ yeast cells transformed with a *CEN*-based plasmid containing the *SAC1*(-100/-1)-GFP fusion construct were grown to early log phase at 30°C. Cell extracts were analyzed by SDS-PAGE and immunoblotting using anti-GFP and anti-glucose-6-phosphate dehydrogenase (Zwf1p) antibodies. **(C) **Quantitation of relative GFP expression levels. Data are from at least three independent experiments (+/-SE).

To identify the regulatory elements that are required for *SAC1 *gene transcription in the yeast *Saccharomyces cerevisiae*, we generated a reporter construct to examine *SAC1 *promoter activity. A genomic region comprising 500 bp upstream of the *SAC1 *open reading frame (*SAC1-500/-1*) was fused to the gene encoding green fluorescent protein (GFP) (Fig. 1A). The activity of the *SAC1(-500/-1) *5'-UTR was then determined by monitoring intracellular GFP levels (Fig. 1B). Yeast cells in which the wild-type copy of *SAC1 *was deleted, showed a five-fold elevated expression from the reporter construct (Fig. 1B, C). The phosphatase-deficient *sac1-8 *mutant caused a similar degree of upregulated *SAC1-GFP *reporter activity (Fig 1D), suggesting that the *SAC1 *promoter is regulated by a mechanism that responds to a loss of Sac1p enzyme activity. To identify essential elements within the *SAC1(-500/-1) *5'-UTR, we constructed a series of truncations within this region and assayed promoter activity (Fig 2A). Elimination of a 150-bp fragment containing a putative TATA box element (bp -50 to -46) within the 5'-UTR abolished expression (*SAC1(-500/-150)*, Fig. 2A). Further truncations led to the discovery of a 100-bp element directly upstream of the *SAC1 *open reading frame that is necessary for promoter activity (Fig 2A). Significantly, the *SAC1(-100/-1) *minimal promoter was not only essential for gene transcription, but also sufficient for producing an elevated expression response in a *sac1*Δ background (Fig. 2B, C).

**Figure 3:**
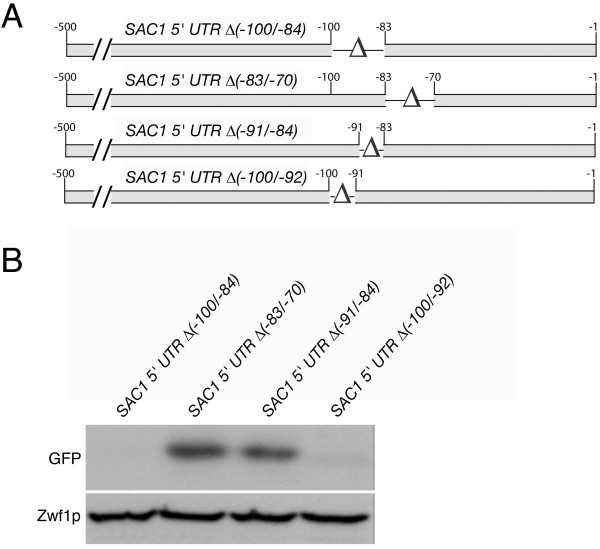
**Identification of a 9-bp element critical for *SAC1 *promoter activity**. **(A) **Diagram depicting deletion constructs. The constructs were fused to the open reading frame of GFP in a *CEN*-based vector. **(B) **Expression of the GFP reporter. The respective plasmids were introduced into a wild-type strain background and promoter activity was determined by measuring relative GFP expression levels. Cell extracts were analyzed by SDS-PAGE and immunoblotting using anti-GFP and anti-glucose-6-phosphate dehydrogenase (Zwf1p) antibodies.

To further investigate *SAC1 *promoter elements, we constructed additional deletions within the *SAC1(-500/-1) *5'-UTR and tested the individual deletion mutants in the GFP reporter expression assay (Fig. 3A). The deletion of base pairs from position -100 to -83 resulted in a significant loss of transcriptional activity, while removal of similar-sized fragments at either side of this region had no effect on promoter activity (Fig. 3B). Two additional deletion mutations uncovered a region consisting of the 9-bp motif ACCAGAGGT ranging from position -100 to -92, which is indispensable for expression (Fig. 3B). Further analysis using the *Saccharomyces cerevisiae *promoter database (SCPD) [12] showed that this motif does not overlap with any known recognition site for DNA-binding factors.

### *SAC1 *expression is regulated independent of inositol levels and ER stress

**Figure 4:**
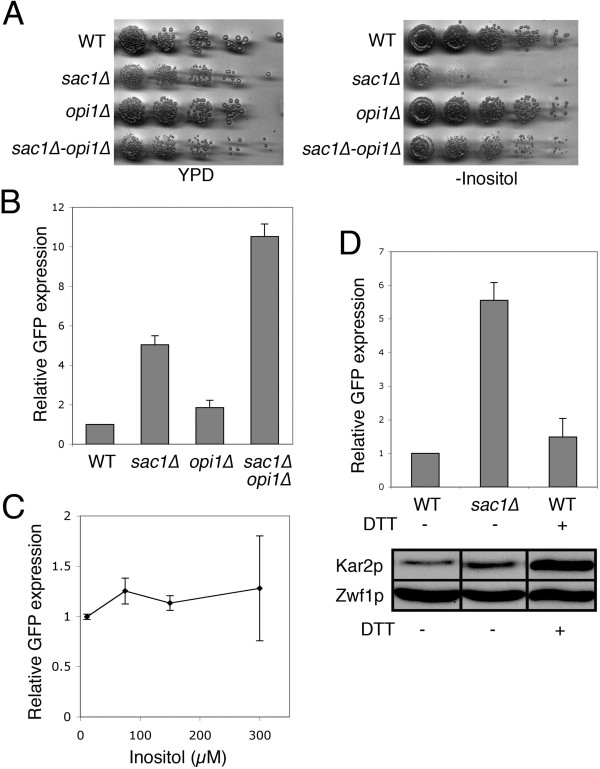
***SAC1 *expression is independent of inositol levels and ER stress**. **(A) **Analysis of cell growth in *sac1*Δ and *opi1*Δ mutants. Cells were grown at 30°C, plated in 5-fold serial dilutions starting with a density of 10^7 ^cells/ml on rich growth medium (YPD) or on inositol-free medium and incubated for 3 days. **(B) ***SAC1 *promoter activity in *opi1*Δ mutants. Cells were transformed with a *CEN*-based plasmid containing the *SAC1*(-500/-1)-GFP fusion construct and grown to early log phase at 30°C. Cell extracts were analyzed by SDS-PAGE and immunoblotting. Relative GFP expression levels were quantified. Data are from at least three independent experiments (+/-SE). **(C) **Influence of inositol on *SAC1 *promoter activity. *sac1*Δ cells transformed with a *CEN*-based plasmid containing the *SAC1*(-500/-1)-GFP fusion construct were grown in media containing a range of inositol concentrations. Relative GFP expression levels were quantified as above. Data are from at least three independent experiments (+/-SE). **(D) **Influence of ER stress on *SAC1 *promoter activity. Wild-type and *sac1*Δ cells transformed with a *CEN*-based plasmid containing the *SAC1*(-500/-1)-GFP fusion construct were cultivated in media with or without 7 mM DTT. Cell extracts were analyzed by SDS-PAGE and immunoblotting using anti-GFP, anti-glucose-6-phosphate dehydrogenase (Zwf1p), and anti-Kar2p antibodies. Relative GFP expression levels were quantified. Data are from at least three independent experiments (+/-SE).

Deletion of *SAC1 *causes specific changes in cellular phosphoinositide levels and induces characteristic cellular defects [13-16]. These in turn may indirectly affect *SAC1 *expression. For example, *sac1 *mutants are inositol auxotrophs, characterized by their inability to grow on inositol-depleted culture medium [17]. Changes in inositol concentrations modulate the expression of many phospholipid biosynthetic enzymes [18]. This regulation involves binding of the transcription factor complex Ino2p/Ino4p to one or several UAS_INO _motifs in the promoter region of relevant genes [18]. Although the *SAC1(-500/-1) *region does not contain a canonical UAS_INO _motif, a negative regulation of *SAC1 *expression by inositol would be consistent with the requirement of a functional Sac1p for growth at low-inositol conditions. To examine whether *SAC1 *promoter activity is regulated through inositol levels, we created a *sac1Δopi1*Δ double mutant. The *OPI1 *gene encodes a negative regulator of inositol and phospholipid biosynthesis and represses activity of *INO2/INO4*-mediated transcription [18]. Elimination of *OPI1 *causes overproduction and excretion of inositol [7,19,20]. Deletion of *OPI1 *in a *sac1*Δ background rescued the growth defect on inositol-free medium (Fig. 4A) but continued to display other *sac1*Δ-specific phenotypes (data not shown). However, expression of the GFP reporter from the *SAC1(-500/-1) *region was not attenuated but enhanced in the *sac1Δopi1*Δ double mutant (Fig. 4B). Yet, the elevated *SAC1(-500/-1) *promoter activity in a *sac1*Δ background did not respond to increasing concentrations of inositol in the growth medium, ruling out the possibility that *OPI1 *deficiency in a *sac1*Δ background simply stimulates *SAC1 *expression by increasing the cellular inositol concentrations. The *sac1Δopi1*Δ double mutant showed also elevated GFP expression from the minimal *SAC1(-100/-1) *promoter (data not shown). Because the *SAC1(-500/-1) *region contains no UAS_INO _motif it remains unclear how *opi1 *deficiency further enhances the expression from this promoter. Combined, these results suggest that inositol is not a regulator of *SAC1 *expression.

Sac1p plays an important role in ER-function by promoting ATP uptake and oligosaccharide biosynthesis [11,15]. Disruption of *SAC1 *induces ER stress and causes constitutive activation of the unfolded protein response (UPR) [15]. To test directly whether *SAC1 *expression is controlled by the UPR, we induced ER stress by treating cells with the reducing agent dithiothreitol (DTT) [21]. While DTT triggered a substantial increase in the cellular levels of the ER chaperone Kar2p (Fig. 4D), expression from the *SAC1(-500/-1) *5'-UTR did not change significantly (Fig. 4D). This result eliminates the possibility that *SAC1 *expression is under control of the UPR. *sac1 *mutants also display defects in actin cytoskeletal arrangement and are sensitive to drugs such as caffeine and Calcofluor White (CFW) [13,14]. However, treating cells with CFW, an agent causing cell wall defects and thus activating the cell integrity pathway, had no obvious effect on *SAC1 *expression (data not shown).

### Intracellular levels of PI(4)P correlate with *SAC1 *promoter activity

**Figure 5:**
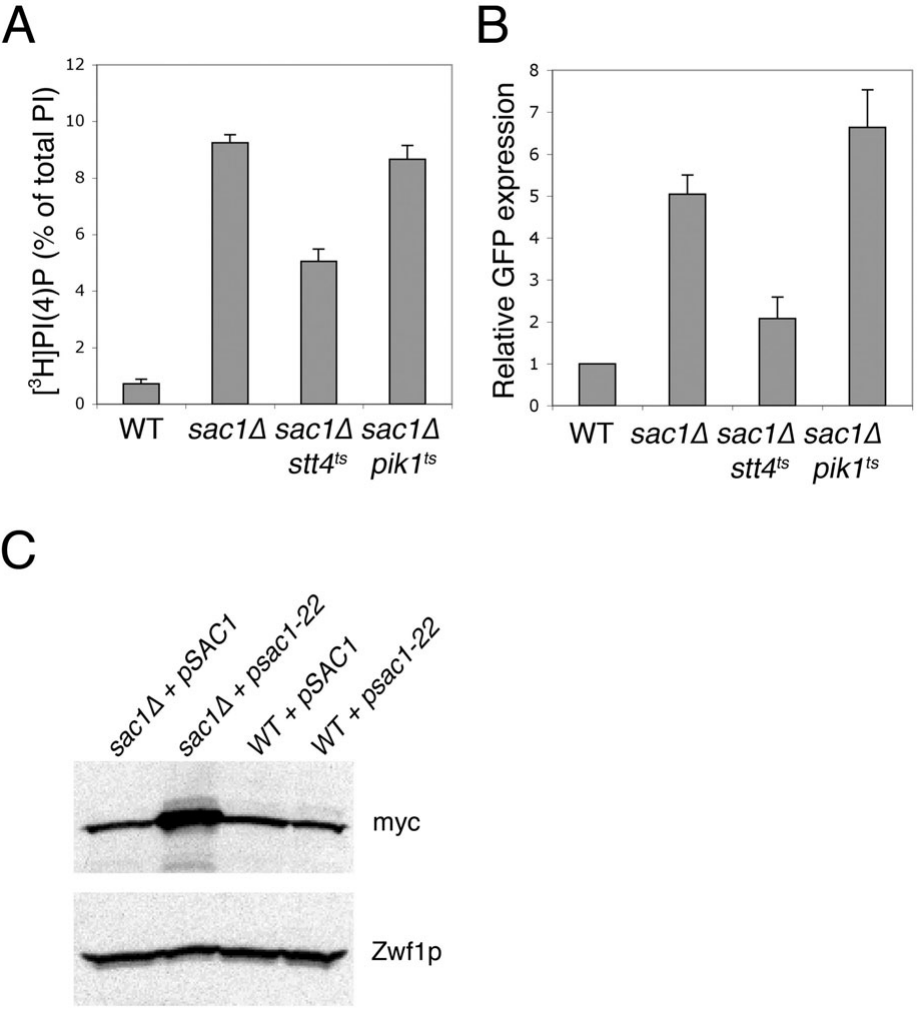
***SAC1 *expression responds to changes in PI(4)P levels**. **(A) **PI(4)P levels in *sac1*Δ and *sac1*Δ PI 4-kinase double mutants. Yeast cells were grown at 33°C and labeled with [^3^H]myo-inositol. Phosphoinositides were extracted, deacylated and quantified by HPLC. Data are from three independent experiments (+/-SE).**(B) ***SAC1 *promoter activity in *sac1*Δ and *sac1*Δ PI 4-kinase double mutants. Yeast cells were transformed with a *CEN*-based plasmid containing the *SAC1*(-500/-1)-GFP fusion construct and grown to early log phase at 33°C. Cell extractswere analyzed by SDS-PAGE and immunoblotting. Relative GFP expression levels were quantified. Data are from at least three independent experiments (+/-SE). **(C) **Correlation of increased Sac1 protein levels and PI(4)P phosphatase deficiency. Wild-type and *sac1*Δ yeast expressing either a myc-tagged wild-type Sac1p or phosphatase-deficient mutant myc-Sac1-22p from the *SAC1(-500/-1) *promoter were grown to early log phase at 30°C. Cell extracts were analyzed by SDS-PAGE and immunoblotting using anti-GFP and anti-glucose-6-phosphate dehydrogenase (Zwf1p) antibodies.

Disruption of *SAC1 *results in pleiotropic changes in cellular phosphoinositide levels. *sac1*Δ cells show a 2-fold elevation in PI(3)P levels and a moderate decrease in PI(4,5)P_2 _[13,22]. The most dramatic phenotype is an approximate 10-fold elevation in PI(4)P levels [13,22]. In proliferating cells, Sac1p is required for confining a PI(4)P pool generated by the PI 4-kinase Stt4p to the plasma membrane. During starvation, Sac1p translocates from the ER to the Golgi and eliminates Golgi PI(4)P, which is generated by the PI 4-kinase Pik1p [13,22]. To examine whether the upregulated activity of the *SAC1 *promoter responds to alterations in one of these PI(4)P pools, we introduced temperature-sensitive mutant alleles of *stt4 *and *pik1 *into a *sac1*Δ background. Both Pik1p and Stt4p are essential for cell growth and it was shown previously that *stt4*^*ts *^or *pik1*^*ts *^strains show impaired PI(4)P biosynthesis and reduced viability at semi-permissive temperatures above 25°C [8,23]. In a *sac1Δstt4*^*ts *^strain cultivated at 33°C, the excess PI(4)P levels were largely reduced, whereas PI(4)P levels in the *sac1Δpik1*^*ts *^strain remained elevated at this temperature (Fig. 5A). These results are consistent with previous reports confirming that Sac1p controls mainly Stt4p-generated PI(4)P during normal cell growth [8,10]. As shown in Fig. 5B, the relative activity of the *SAC1(-500/-1) *5'-UTR correlates significantly with the respective changes in PI(4)P in these mutant strains. This result indicates that expression from the *SAC1 *promoter responds to the levels of intracellular PI(4)P. To confirm that the PI(4)P-induced activity of the *SAC1 *promoter is reflected by increased Sac1p protein levels, we used the *sac1-22 *allele, encoding a phosphatase-deficient Sac1 protein [24]. In wild-type cells a myc-tagged version of Sac1-22p was expressed at the same level as Sac1p. In contrast, expression in a *sac1*Δ strain induced a significant increase in myc-Sac1-22p protein levels whereas protein levels of myc-tagged wild-type Sac1p remained unchanged (Fig. 5C). In summary, these results indicate that Sac1p protein levels respond to a rise in intracellular PI(4)P.

## Discussion

In yeast, many enzymes required for phospholipid biosynthesis show a common pattern of transcriptional regulation [5]. Soluble and membrane-bound precursors for phospholipid biosynthesis such as inositol, choline and phosphatidic acid play a major role in this regulation [6,7]. In contrast, little is known about the transcriptional regulation of enzymes controlling the cellular levels of the phosphorylated derivatives of these phospholipids. While Sac1p function is essential when yeast cells are deprived of inositol [17], the expression of *SAC1 *is not regulated by inositol itself. Instead Sac1p protein levels respond to the cellular levels of PI(4)P, which is the major substrate of this lipid phosphatase. PI(4)P is concentrated in distinct intracellular pools that have diverse yet essential cellular functions such as in regulating membrane trafficking and actin cytoskeletal organization [8,10,11]. In proliferating cells, Sac1p is responsible for turning over the PI(4)P that is generated by the PI 4-kinase Stt4p [25]. We find that alterations in this Stt4p-specific PI(4)P pool are mechanistically linked to the control of *SAC1 *expression.

Membrane homeostasis and organellar traffic both rely on precisely regulated phosphoinositide gradients. In growing cells, Sac1p plays an important role in preventing random equilibration of PI(4)P at intracellular membranes, a phenotype commonly observed in *sac1 *mutants [8,9]. Linking *SAC1 *expression to the levels of PI(4)P ensures that sufficient levels of the lipid phosphatase are continuously available to fulfill this task. Analysis of promoter elements required for this regulation revealed the partially palindromic 9-bp motif in the 5'-UTR of *SAC1 *that is critical for expression. Partial palindromic sequences have also been found in other cis-acting promoter elements [26]. However, queries in the *Saccharomyces cerevisiae *promoter database (SCPD) indicate that the ACCACAGGT element does not overlap with any known consensus sequence for DNA binding proteins and therefore represents a novel motif. *SAC1 *promoters in higher eukaryotes have not yet been defined and it remains to be seen whether expression of the mammalian SAC1 homologs is regulated via a similar element.

*sac1 *mutants display accumulation of PI(4)P at the nuclear envelope and it is possible that nuclear phosphoinositides activate or recruit hitherto uncharacterized factors required for transcription. Recent reports indicated that phosphoinositides play important roles inside the nucleus and nuclear phosphoinositide-binding proteins have been discovered [27,28]. While our results support the idea that PI(4)P is a direct regulator of *SAC1 *gene expression, it is also possible that a metabolite downstream of PI(4)P is the actual signal transducer. PI(4)P can be rapidly converted to PI(4,5)P_2 _by the PIP kinase Mss4p [29,30]. However, *sac1 *mutant strains do not show elevated PI(4,5)P_2 _levels [22] and it is therefore unlikely that PI(4,5)P_2 _is directly involved in this regulation. Another potential mechanism could involve soluble inositol phosphate species. Both PI(4)P and PI(4,5)P_2 _can be hydrolyzed by phospholipase C giving rise to inositol-1,4-bisphosphate and inositol-1,4,5-trisphosphate respectively [31]. These soluble signal transducers can be further phosphorylated in the nucleus where they are involved in transcriptional control and mRNA export [32,33]. It remains to be determined whether these molecules play a role in regulating *SAC1 *expression and identifying the additional components of this signaling mechanism awaits further investigations.

## Conclusion

This study characterizes a promoter element required for regulated expression of the lipid phosphatase Sac1p in yeast. This enzyme controls the distinct intracellular pools of PI(4)P required for membrane traffic and homeostasis. Distinct from phospholipid biosynthetic enzymes, whose expression is largely regulated by small soluble phospholipid precursors, the activity of the *SAC1 *promoter correlates with the intracellular levels of PI(4)P. We propose that the precise control of Sac1 protein levels by the membrane concentration of its major substrate ensures proper maintenance of organelle-specific phosphoinositide gradients.

## Methods

### Strains, reagents, and other procedures

Plasmids, strains and DNA primers are listed in Table 1 and 2. *Saccharomyces cerevisiae *strains were grown in standard yeast extract/peptone/dextrose (YPD) media or Hartwell's complete media (HC). The *OPI1 *disruption cassette was created by PCR, using the primers Opi1KOfwd, Opi1KOrev and the vector pRS413 [34] as template. The PCR product was transformed into ATY201 and STY39. Antibodies against glucose-6-phosphate dehydrogenase (Zwf1p, working dilution 1:100,000) and GFP (working dilution 1:2,400) were purchased from Sigma-Aldrich (St Lois, MO). [^3^H]myo-inositol was purchased from PerkinElmer (Wellesley, MA). SDS-PAGE and immunoblotting were performed as described [35].

**Table 1:** Plasmids and yeast strains

	Genotpye	Origin
Plasmid		
pGK25	*CEN ARS URA3 GFP*	[24]
pGK26	*CEN ARS URA3 SAC1 5' UTR (-500/-1)-GFP*	[24]
pAK29	CEN ARS URA3 SAC1 5' UTR (-242/-1)-GFP	This study
pAK30	*CEN ARS URA3 SAC1 5' UTR (-170/-1)-GFP*	This study
pAK34	*CEN ARS URA3 SAC1 5' UTR (-500/-150)-GFP*	This study
pAK36	*CEN ARS URA3 SAC1 5' UTR (-125/-1)-GFP*	This study
pAK38	*CEN ARS URA3 SAC1 5' UTR (-83/-1)-GFP*	This study
PAK40	*CEN ARS URA3 SAC1 5' UTR (-114/-1)-GFP*	This study
pAK42	*CEN ARS URA3 SAC1 5' UTR (-100/-1)-GFP*	This study
pAK47	CEN ARS URA3 SAC1 5' UTR Δ(-100/-84)-GFP	This study
pAK48	*CEN ARS URA3 SAC1 5' UTR Δ(-83/-70)-GFP*	This study
pAK49	*CEN ARS URA3 SAC1 5' UTR Δ(-91/-84)-GFP*	This study
pAK50	*CEN ARS URA3 SAC1 5' UTR Δ(-100/-92)-GFP*	This study
Strain		
ATY201	MATα*trp1-delta901 leu2-3,112 his3-delta200 ura3-52 lys2-801 suc2-delta9 can1::hisG*	[36]
STY39	MATa *trp1-delta901 leu2-3,112 his3-delta200 ura3-52 lys2-801 suc2-delta9 can1::hisG sac1::TRP*	[8]
STY40	MATa *leu2-3, 112 ura3-52 his3-delta200 trp1-delta901 lys2-801 suc2-delta9 sac1::TRP1 stt4::HIS3 pSTT4-4 (LEU2 CEN6 stt4-4)*	[8]
STY47	*Pik1::ADE2-1 sac1::TRP YEplac181::pik1-12*	[8]
PMY434	MATα*trp1-delta901 leu2-3,112 his3-delta200 ura3-52 lys2-801 suc2-delta9 can1::hisG opi1::HIS3*	This study
PMY435	MATa *trp1-delta901 leu2-3,112 his3-delta200 ura3-52 lys2-801 suc2-delta9 can1::hisG sac1::TRP opi1::HIS3*	This study

### Generation of *SAC1 *promoter constructs

Fragments of the *SAC1 *5' UTR were amplified by PCR (see Table 2 for oligonucleotide sequences), ligated into pGEM-T Easy Vector (Promega, Madison, WI) and subcloned into pGK25 [24] using *NotI *and *XhoI *restriction sites. Deletion mutations within the *SAC1(-500/-1) *5'-UTR were generated by mutating pGK26 using the QuikChange II Site-Directed Mutagenesis Kit (Stratagene, La Jolla, CA).

**Table 2:** Oligonucleotides

Primer	Sequence
Sac1(-500)fwd	TTGCGGCCGCACAGCTACCACATCCCTGAC
Sac1(-242)fwd	TTGCGGCCGCCCAAGCCTCGCTCCTATTGT
Sac1(-170)fwd	TTGCGGCCGCCTGCACTACTGCTTACCCACA
Sac1(-125)fwd	TTGCGGCCGCGAAGTTGAAAAGGCAAGGGA
Sac1(-114)fwd	TTGCGGCCGCGGCAAGGGAAAAATACCACA
Sac1(-100)fwd	TTGCGGCCGCACCACAGGTTTAGATAAGGA
Sac1(-83)fwd	TTGCGGCCGCGGAAATAGGAGAAAGGATTAG
Sac1(-1)rev	GGCTCGAGATCTAGACGAGAAAATATACG
Sac1(-150)rev	GGCTCGAGTGTGGGTAAGCAGTAGTGCAG
Sac1Δ(-100/-84)fwd	GAAAAGGCAAGGGAAAAATGGAAATAGGAGAAAGG
Sac1Δ(-100/-84)rev	CCTTTCTCCTATTTCCATTTTTCCCTTGCCTTTTC
Sac1Δ(-83/-70)fwd	CCACAGGTTTAGATAAGGATTAGAAACCATATCC
Sac1Δ(-83/-70)rev	GGATATGGTTTCTAATCCTTATCTAAACCTGTGG
Sac1Δ(-91/-84)fwd	GGGAAAAATACCACAGGTGGAAATAGGAGAAAGG
Sac1Δ(-91/-84)rev	CCTTTCTCCTATTTCCACCTGTGGTATTTTTCCC
Sac1Δ(-100/-92)fwd	GGCAAGGGAAAAATATTAGATAAGGAAATAGG
Sac1Δ(-100/-92)rev	CCTATTTCCTTATCTAATATTTTTCCCTTGCC
Opi1KOfwd	CATATCAGGCCAGAACGTGGCATTTTGTTTACAGTGCTGAAGATTGTACTGCTGAGAGTGCAC
Opi1KOrev	AACTATATTATTCCGTATAATATTATTACTGGTGGTAATGCTGTGCGGTATTTCACACCG

### Quantification of protein levels

Cells expressing GFP under the control of *SAC1 *5'-UTR constructs were grown in Hartwell's complete media (HC) supplemented with the appropriate amino acids and harvested in early logarithmic growth phase. 5 OD cells were collected, washed in water and resuspended in 200 μl 2× Laemmli buffer and 200 μl glass beads. Lysates were prepared by vortexing for one minute. Supernatants were boiled for 5 minutes and analyzed by SDS-PAGE and immunoblotting. Protein levels were measured by determination of band size and band density using NIH Image software (version 1.62). Protein amounts of GFP were normalized against protein amounts of glucose-6-phosphate dehydrogenase.

### Lipid analysis

Since *sac1 *mutants are inositol auxotrophs, yeast cells were cultivated in 5.5 μM inositol prior to and during the labeling procedure. Early log phase cells were incubated with 10 μCi/ml myo- [^3^H]inositol for 2–3 doubling times. Labeling, extraction and deacylation of lipids was performed as described previously [36]. HPLC analysis of glycerophosphoinositols was carried out on a 250 × 4.6-mm Partisil SAX column (Whatman, Florham Park, NJ) using a Jasco HPLC system equipped with an LB 508 Radioflow detector (Berthold, Bad Wildbach, Germany). Elution and quantification of glycerophosphoinositols were performed as described [36].

## Authors' contributions

AK performed the experiments, contributed to the experimental design, and helped in the writing of this manuscript. GK collaborated in protein expression analyses. PM coordinated this study, provided its conceptual basis, participated in experimental design and wrote the manuscript. All authors read and approved the final manuscript.
